# Extending “Continuity of Care” to include the Contribution of Family Carers

**DOI:** 10.5334/ijic.2545

**Published:** 2017-06-27

**Authors:** Cecilia Wong-Cornall, John Parsons, Nicolette Sheridan, Timothy Kenealy, Allie Peckham

**Affiliations:** 1 School of Nursing, School of Population Health, University of Auckland, Private Bag 92019, Auckland 1142, NZ; 2 School of Nursing, University of Auckland, Private Bag 92019, Auckland 1142, NZ; 3 The Institute of Healthy Ageing, Waikato District Health Board, NZ; 4 School of Nursing, Faculty of Medical and Health Sciences, University of Auckland, Private Bag 92019, Auckland 1142, NZ; 5 School of Medicine, University of Auckland, Private Bag 92019, Auckland 1142, NZ; 6 Institute of Health Policy, Management and Evaluation, University of Toronto, CA

**Keywords:** family caregiving, informal care, chronic conditions, older people, continuity of care, primary health care

## Abstract

**Background:**

Family carers, as a “shadow workforce”, are foundational to the day-to-day integration of health service delivery for older family members living with complex health needs. This paper utilises Haggerty’s model of continuity of care to explore the contribution of family carers’ to the provision of care and support for an older family member’s chronic condition within the context of health service delivery.

**Methods:**

We analysed data from interviews of 13 family carers in a case study of primary health care in New Zealand – a Maori Provider Organisation – to determine the alignment of family caregiving with the three levels of continuity of care (relational continuity, informational continuity, and management continuity).

**Results:**

We found alignment of family caregiving tasks, responsibilities, and relationships with the three levels of continuity of care. Family carers 1) partnered with providers to extend chronic care to the home; 2) transferred and contributed information from one provider/service to another; 3) supported consistent and flexible management of care.

**Discussion:**

The Maori Provider Organisation supported family carer-provider partnership enabled by shared Maori cultural values and social mandate of building family-centred wellbeing. Relational continuity was the most important level of continuity of care; it sets precedence for family carers and providers to establish the other levels – informational and management – continuity of care for their family member cared for. Family carers need to be considered as active partners working alongside responsive primary health care providers and organisation in the implementation of chronic care.

## Introduction

Family carers are a “shadow workforce” that provide crucial day-to-day support to family members with complex health conditions. They are vital to effective chronic care management [1]. Family carers help link family members with health care services, including primary health care and general practice. They can translate and explain health information [2,3], navigate and coordinate services [4,1], and advocate for their family members’ needs and rights [5,6,7,8]. Acting as integrators, family carers meet gaps in formal health and social services, especially for older adults [1,4,5,9], and support care that extends into homes [10,11].

Many carers carry out complex and difficult tasks without recognised training, and often with little support. The informal status of family carers – they are not acknowledged by formal health services and are mostly unpaid – has led to ambiguity about their role and capabilities. It is, in part, this uncertainty that has led family carers, and those they care for, to experience fewer opportunities than health care providers to contribute to decisions about care and treatment, coordination of services, and provision of self-management support [4,12,13]. We suggest family carers play a fundamental role in supporting patient self-management and should be recognised as partners in an integrated model of care [14,15].

It is well documented in the patient and carer literature that high-quality care at home relies upon strong relationships between health professionals and family carers who advocate for care recipients [5,6,7,12], support access to information, and undertake training related to caregiving tasks [2,3]. Communication between clinicians and patients with chronic illnesses and their family carers “characterised by shared understandings and respect leading to participatory decision making” can positively influence health outcomes [16]. These authors contend health providers have a primary responsibility to facilitate patient and family carer engagement in care. Other commentators suggest health providers can reinforce a family carer’s central role as case-manager [1,4]. Kodner and Spreeuwenberg (2002) [15] suggest successful health and social service integration occurs only if family carers and patients are involved in the planning and implementation of care. However, empirical findings of family carer-provider (usually nurses) relationships highlighted differences between the values and expectations of the two workforces that limited the willingness from both sides to partner in chronic care [17,18,19]. There is a lack of discussion in the literature on how informal carers – such as unpaid family carers – partner with providers and services to support integrated care.

Haggerty (2003) [20] has defined continuity of care to include three complementary domains – relational, informational and management of care – all within the context of provider interactions with patients, supported by provider organisations. Relational continuity supports an ongoing relationship between providers and patient/family bridging past, present and future care. Informational continuity is the transfer of information from past events and personal circumstances to ensure the appropriateness of current care for the patient and family. Management continuity is the consistent management of care across services through shared management between patient/family and provider and flexibility in responding to changes in a patient or family’s needs. We will argue that family carers extend the functions of continuity beyond the role of provider and organisation, in ways that, to date, have been largely ignored in the literature. The aim of this paper is to examine family carer’s contribution to continuity of care in alignment with Haggert’s model. It will present perspectives of family carers caring for older family members in a Maori community-based primary healthcare organisation.

## Methods

We draw on family carer data from a New Zealand case study nested within an international research project investigating community-based primary health care [21]. The international study collect data across three levels – macro (policy), meso (organisation and provider) and micro (patient and carer) – from identified cases of innovative models of integrated community-based primary healthcare across New Zealand, Ontario, and Quebec. The case study organisation selected for this paper is a not-for-profit Maori health provider organisation located within an area of approximately 20,000 residents of whom about 5,000 are of Maori descent. This organisation provides no-cost primary health care services in urban, semi-rural, and rural clinics run by a nurse practitioner, nurse or a general practitioner for those enrolled with the organisation [22].

Participants were purposively sampled. They were eligible if they were 16 years of age or older at the time of the interview and were the primary or significant carer of an older family member enrolled as a patient with the Maori Provider Organisation. They assisted that family member with day-to-day functions and the self-management of their chronic health conditions. ‘Older’ family member receiving care who were of Maori or Pacific descent were aged 50 years and older and those who were non-Maori or non-Pacific were aged 65 years and older. The age definition for ‘older’ people in vulnerable populations such as Maori is lower because they have a shorter life-expectancy and experience conditions associated with older age earlier in their life-course [23].

Fifteen family carers responded to recruitment posters in the clinics of the Maori Provider Organisation and gave the clinic receptionist their contact details to be passed on to the research team. A brief telephone conversation with a researcher confirmed eligibility of 13 family carers to participate in the study.

Thirteen semi-structured interviews were conducted by CWC with family carers between February and March 2015. Five topics were covered in the interviews: personal details and carer information; personal perspectives on care; care and assistance; health services; health and wellbeing. Interviews included the Carer Reaction Assessment [24], Cultural Justification for Caregiving Scale [25], Activities for Daily Living and Instrumental Activities for Daily Living Scale [26], and Hua Oranga, a Maori mental health assessment scale [27] to initiate and guide discussions around family carers’ experience and engagement with community-based primary healthcare services. The interviews ranged from between 60 to 90 minutes. The interviews were transcribed verbatim. CWC, JP, and NS analysed the interview transcripts using a deductive approach. The descriptive criteria of Haggerty’s model of continuity of care were used as an analysis framework to explore family carer’s narratives. TK reviewed transcripts and data interpretation, further validating processes surrounding data analysis.

## Results

Thirteen family carers ranging from 35 to 75 years of age were interviewed. Of these, 11 were female, and all were caring for older family members with multiple complex health conditions. Some carers were also managing their own chronic health conditions. Most were full-time carers of family members, although some carers juggled this role alongside paid employment. Ten participants identified to be Maori, 2 identified to be New Zealand European, and 1 had mixed Maori and European ethnicity. The New Zealand European family carers were married to and caring for their Maori spouse; one was fluent in Maori language. All carers were the main family member orchestrating care for their older family members in a multigenerational context where extended ‘whanau’ (a Maori term referring to an extended family or community of related families who share genealogical, physical, emotional, and spiritual connections) were partially involved in the wider familial caregiving structure. Household income was low in 10 homes with 7 receiving financial benefits from Work and Income New Zealand. Families lived in a mix of suburban and semirural or rural locations, which often meant that transportation (most commonly the family car) was required to access the closest amenities, such as a supermarket, pharmacist, or petrol station. The characteristics of the participants are summarised in Table 1.

**Table 1:** Participant characteristics.


**Gender**	
Male	2
Female	11
**Ethnicity**	
Maori	10
NZ European	2
Mixed (Maori and European)	1
**Age Range**	
35–49	3
50–64	8
65–74	1
75+	1
**Familial relationship with family member cared for**	
Wife	5
Husband	1
Daughter	6
Son	1
**Currently living with family member cared for**	
Yes	10
No	2
Family member deceased	1
**Total**	**13**


### Relational continuity of care

The ‘family carer – provider’ relationship was foundational to enabling continuity of relationship between provider and patient and it appeared that the Maori Provider Organisation staff understood the importance of this relationship. The high level of engagement family carers had with the general practitioner (GP), nurse practitioner (NP), nurse, community worker, and other allied staff facilitated care that extended into patients’ homes. Family carers who had developed partnerships with a specific GP or NP for example shared an advocacy role where both family carer and provider championed patient access to health services and welfare benefits. Carers described the process of working with the GP and NP as one that built knowledge, confidence, and skills in caregiving. They reported that ‘teaming-up’ with a clinician helped them overcome barriers to accessing not only primary care but also specialist hospital care. Repeatedly we observed an approach that was unified, trusting, and a consequence of multiple interactions between providers and family carers. Well established relationships reflected a higher level of engagement than newer relationships with family carers and family members who had more recently settled in the district and enrolled with the provider.

*“[GP] and I are pushing at the moment for [husband] to go down to Auckland [hospital], but in order to do that you’ve got to go through the specialist up here to refer you down there.”* (Female carer, aged 50–64)

*“I talked to [NP] and explained to her about my emotions, and same with Mum, [NP] she is able to [say] ‘Maybe this organisation can help you or… here is the phone number, give them a call. We can look at subsidising it.’”* (Female carer, aged 35–49)

The Maori Provider Organisation aimed to support continuity of care across health and social sectors to reflect the wide-ranging needs of multigenerational families, including older family members. Some family carers were specifically aware of the alignment of their cultural and family values to those of the organisation. The “patient” was central to family carer-provider relationship. In all of the cases, the family carer-provider relationship was built on a strong patient-provider relationship. The GP or NP worked closely with the patient and this work extended to inclusion of the family carer and other family members. Family carer and provider understood that they shared a common goal to support the patient and to improve the extended family’s wellbeing. Family carers perceived that the Maori Provider Organisation understood their approach to health and wellbeing, which was deeply rooted in a Maori, collectivist model that emphasised family wellbeing. These values are embedded within the actions of Maori health provider organisations and embodied within the spirit and policy of Whanau Ora, a cross-government indigenous health initiative driven by Maori family-centred cultural values [28]. The Maori Provider Organisation staffs were instrumental in supporting families to develop stronger relationships with other health and social service providers. Clinicians went beyond caring for their patients’ health conditions to caring for other family members revealing a family-centred understanding of chronic care management. Clinical and non-clinical staff supported family carers to connect with the Maori Provider Organisation at a service level as well as an interpersonal level.

*“I’m hoping that places like [Maori Provider Organisation] are able to set a really good example of how people should… what people need… And, to be a bit more sympathetic on how Maori are with families.”* (Female carer, aged 35–49)

*“They [Maori Provider Organisation] are very finger on the pulse way of looking after. Even if they are not here in person, they know what is going on. They are very connected. And when they do come, they… yeah. It’s just a very whanau feeling about their service.”* (Male carer, aged 50–64)

### Informational continuity of care

Family carers filled an active role in supporting the transfer of health information from one provider to another and from one health care event to another. Most family carers had become a depository of their family member’s health information. They recorded and recalled medical notes and anecdotal information of their family member’s past health care events, history of diseases and current medical treatments including their up-to-date prescribed medications. They helped fill in gaps in their family member’s health records at the commencement of the provision of new services. Some family carers identified their ability to pass on health information to be particularly important at times of emergency, such as when their family member became severely ill and was unable to respond to clinicians at the hospital or from emergency services. Similarly, family carers provided feedback to GP or NP to update them about their family member’s experiences outside of primary health care, most often after hospitalisation. This communication process allowed the GP or NP to gain comprehensive knowledge of the patient’s health experiences beyond the medical aspects of their care. Family carers provided an important linkage for maintaining and updating their family member’s centralised health record where relevant health information will be pass on to inform future health events and interventions.

*“Oh yeah, I can ring [GP] day or night. And [GP]’s making me [call him after husband’s treatment at the hospital]… because he knows that I’ve been a caregiver for years, and he knows that when I come to him for a question, it’s not bullshit. It’s for real.”* (Female carer, aged 50–64)

Most family carers felt they have the best knowledge of their family member’s needs through accumulated personal and caregiving experiences. They understood their family member’s health care preferences, such as when or how their family member would like services to be delivered; important values that extended to health care, inclusive of cultural beliefs and familial traditions; and circumstantial contexts related to care, such as the social and financial needs of the family member or the wider family. Many family carers actively supported their family member to inform and explain their personal needs to service providers. Some family carers identified that their role in transferring non-medical information from their family member to the services was necessary because their family member may be living with cognitive or physical impairment that had made communication difficult (for example with dementia or a stroke). Family carers informed and worked with services on behalf of their family member to find solutions that were personally, culturally, and circumstantially appropriate to their needs.

*“Dad prefers Maori, and so they are able to talk to dad in Maori. Um, we had a European lady that came. Um, we had to ask [service provider] if we can have someone that knew Maori so they could relate more, both with mum and dad. But dad prefers it, even mum if you speak Maori. Is not as though they can’t speak English but with dad… yeah, with dad and his state of mind [dementia], he prefers Maori to English.”* (Male carer, aged 50–64)

### Management continuity of care

Most family carers were already managing what they could of the health and social services for their family members in a day-to-day capacity. This is consistent with carer roles in the literature [1,4]. Tasks performed by family carers that improved the integration of services included maintaining treatments plans, scheduling services and appointments, and liaising between health, community and social agencies.

Haggerty (2003, 2008) [20,29] identified consistency and flexibility as two critical components of management continuity in long-term care. Management tasks performed by family carers supported both consistent and flexible management of their family member’s care. Their ability to support management continuity was built on strong relationships with the providers and good informational continuity.

#### Consistency

Family carers reported that the strong partnership they have with providers had allowed them to be part of their family member’s care planning and decision making around treatments and self-management from the beginning of their caregiving journey. For many family carers and their family members, the shared care plan that has been put in place to manage a particular condition had already been adapted into their day-to-day routine and embedded in their relationship with the provider or service. The continued process of sharing recorded information between provider and the family had ensured that the family carer and their family member remained well informed of the care plan and treatments. Family carers readily gave examples of specific care pathways established by the provider and their family member to deal with and treat medical events as they occurred; they were familiar with the details of the pathway, how to carry out the treatments and the expected outcomes by following the care plan. Many family carers and their family members shared a sense of security because they know that the care plan works and is ready to support them in cases of emergencies.

*“Ring [GP] and tell him. He makes an appointment to see the specialist in Auckland that usually takes months. And we got down there to see the specialist, and then they book us in, book her in for the operation. Then we wait again for the op, then take her down for the op. She stays in for a few days. Go down again to pick her up, bring her home. And then she usually got her eye covered for a week. And then we take her back to [GP] to unravel it. Boom, she can see again.”* (Male carer, aged 50–64)

The carer described the care pathway for his wife when she experienced diabetic retinopathy; this care pathway has been used four times at the time of the interview and is in place for future onset of her illness.

#### Flexibility

The established relationship between family carers and providers enabled family carers to willingly feedback changes in their family members’ condition or reactions to medications/treatments immediately. They also reported changes in family circumstances that had a bearing on care. Family carers were pivotal in assisting clinicians to respond earlier by adapting care plans to mitigate health needs.

*“I mean, the biggest thing that I’ve found is that if I need – like, if mum wants a change in something [with health services], I can go to [Maori Provider Organisation] and ask them.”* (Female carer, aged 35–49)

*“Ring the hospital or [Maori Provider Organisation] rings them on our behalf. Yeah, so they would ask “Do you need anything?”, and we would say “Yes, this and that.” And so they will talk to the different people [services and providers] and then we would get a call from them via [Maori Provider Organisation]. So yeah they do um… if there is a need they always connect us with the right people. Or they will ring back to us.”* (Male carer, aged 50–64)

## Discussion/Conclusion

Our findings suggested that family carers actively contributed to continuity of care for their older family members. They provide health information to inform providers of what has happened before, they advocate for their family member’s interest in shared management plan, and they build relationships with providers who will care for their family in the future. Our findings reinforced evidence from family caregiving literature that family carers already complement and extend functions of chronic care often in parallel to formal services – such as patient’s case-management and information sharing [1,4,5,9].

The caregiving situations experienced by family carers from our study were reflective of the challenges described in the literature and other New Zealand studies [8,30]. Carers had a difficult task managing the multiplicity of their roles – juggling the increasing complex needs of their older family member cared for, the wellbeing of other family members (including dependent children), personal health issues, and employment and personal goals. The financial struggles – the extra cost of caregiving paired with existing poverty – faced by most families highlighted the complex needs of the carers, their family, and older family member cared for.

The distinguishing feature of the Maori Provider Organisation as a service for the family carers was their willingness to partner and support the whole family in chronic care. Our findings is congruent with Guthrie et al (2008) [31] who reported that relationships, in this case between provider and the family unit inclusive of the patient and family carer, are crucial for establishing good long-term care. Unlike findings from other studies of family carer-provider relationships where providers either avoided or exploited their engagement with family carers [18,19], family carers and providers in this case mutually sought to work together to support the patient. The organisation understood the important role family carers filled in chronic care management that has largely been invisible in the literature and policies. They also acknowledged the self-management and caregiving challenges that this population faced. The Maori Provider Organisation responded through their services supporting clinicians reach carers in homes. Clinicians would go ‘out of their way’ to support family carers manage care based on medical and personal information over time, in contrast to the reactive culture of health services that respond to medical emergencies as they arise [12]. The Maori Provider Organisation reorganised funding from Whanau Ora government contracts to fund the needs of families caring for older people. Clinicians shared the organisation health and social mandate, family-centred values, and built interpersonal relationships with family carers and their patients. This model of extending and engaging patient, family carers, and the wider family in the chronic care was achievable because it has been consciously supported by the organisation. While this paper described interactions between family carers, patients, and providers within a Maori community-based healthcare context, study findings related to a family-centred service model are potentially transferable to other ethnic minority or cultural groups.

Our study presents the experiences of family carers from one Maori Provider Organisation and we acknowledge this limits any generalisability. However, we suggest learnings from this study are transferable to other frameworks or models of care that are family-centred. The family carers in this case study were clear that case-management was a central component of their role and they sought to build formal partnerships with providers who supported them to achieve their caregiving goals.

A growing body of literature [29,31,32,33], have extended measurements of continuity of care to strongly reflect the perspectives of the patient, particularly patient-provider partnership, but not to the perspectives of family carers. The hitherto-hidden role of family carers which often function in parallel to formal services offers potential insights and partnerships for achieving and improving continuity of care. Goodwin, Dixion, Anderson and Wodchis (2014) [10] found integrated models of chronic care were more likely to be successful when the organisation and providers worked directly with patients and informal carers to support self-management. The current study provided evidence of family carers acting as integrators with providers to enable continuity of care for their older family member. We suggest future studies that investigate and measure continuity of care should include measurement of family carer contribution. Particular measurements should be designed to assess the level of relational engagement between family carers and providers, and the impact of family carer-provider partnerships on informational and management continuity with the aim to shift the reactive culture of health services.
